# Toward a better understanding of the enhancing/embrittling effects of impurities in Nickel grain boundaries

**DOI:** 10.1038/s41598-019-50361-3

**Published:** 2019-10-01

**Authors:** El Tayeb Bentria, Ibn Khaldoun Lefkaier, Ali Benghia, Bachir Bentria, Mohammed Benali Kanoun, Souraya Goumri-Said

**Affiliations:** 1 Laboratoire Physique des matériaux, Université Ammar Telidji de Laghouat; BP 37 G, Laghouat, 03000 Algeria; 2 Physics Department, College of Science, King Faisal University, P.O. Box 400, Al-Ahsa, 31982 Saudi Arabia; 3 College of Science, Physics department, Alfaisal University, P.O. Box 50927, Riyadh, 11533 Saudi Arabia

**Keywords:** Electronic properties and materials, Atomistic models

## Abstract

The fracture path follows grain boundaries (GB) in most metallic system under tensile test. In general, impurities, even in ppm concentration, that segregate to these boundaries can remarkably change materials mechanical properties. Predicting impurities segregation effects in Nickel super-alloys might not be seen as intuitive and perhaps more fundamental understanding is needed. We performed a density functional theory calculation to elucidate the effect of eight light elements (B, C, N, O, Al, Si, P and S) and twelve transition metal elements (Tc, Ti, V, Cr, Mn, Zr, Nb, Mo, Hf, Ta, W, Re) on Nickel ∑5(210) grain boundary formation and its Ni free surface. The effect of impurities was carefully examined by calculating different properties such as segregation, binding and cohesive energies, strengthening/embrittling potency and the theoretical tensile strength. Additionally, we employed the electron density differences and magnetic effects to explain why and how impurities such as B, S, V, Nb, Mn and W affect Nickel ∑5 GB. We used the generated data calculated on equal footing, to develop a fundamental understanding on impurity effect. A clear and strong correlation is found between difference in magnetic moment change between isolated and imbedded impurity atom on one hand and the tensile strength on the other hand. The higher the loss of the magnetic moment, the more the impurity consolidates the GB.

## Introduction

Grain boundaries (GBs) are common type of defects in crystalline solids that strongly affect the physical and mechanical properties of materials. Impurities in ppm concentration in bulk can segregate under high temperature and/or aging process to GBs region at extremely higher concentration^1^, and result in a dramatic reduction/enhancement of the ductility and strength of materials^2^. It has become increasingly important to gain a deep understanding of impurities effects on GB cohesion at the microscopic level for developing new materials. Besides, first principle calculations are promising and cost-effective research strategy widely used in recent years to study the role of segregated impurity atoms in metals and alloys. In the last two decades, numerous efforts have been devoted in determination of impurities impact on nickel grain boundaries using computational methods. The commitment to the GB research topic, was motivated by the industrial challenges facing fabrication and aging of nickel based materials. Also experimental studies were constrained to investigate one impurity alone without the influence of other impurities^3,4^. In the literature, there are mainly four systematic studies on the effect of impurities in Nickel ∑5 GBs based on density functional theory (DFT) calculations^2,5–7^. The first one is by Všianská and Šob^2^, where systematic first-principle study was performed to study the segregation of *sp*-impurities at ∑5(210) (GB) and (210) free surface (FS). Local atomic volumes, interlayer distances and magnetic moments, for both clean and segregated impurities to GB/FS, were analyzed. Furthermore, they calculated the strengthening/embrittling energy according to the Rice–Wang model^8^. The second work was published by Razumovskiy *et al*.^5^, where the segregation and free surface energies of some alloying elements (Ta, Re, Hf, W, Zr) to GB were computed with DFT and considered Hf and Zr as “minor alloying additions”. The third work as achieved by Sanyal *et al*.^7^ using DFT, has determined decohesion properties of Ni ∑5(210) grain boundary with B, C, S, Cr, and Hf dopants. The relative stability of the considered sites was evaluated and the cohesive energies were calculated. The latest study, performed by Razumovskiy *et al*.^6^, investigated the influence of 4d and 5d transition metal alloying elements as well as B, Bi, S and Cr on the GB strength in Ni within the framework of the Rice–Wang approach and by calculating the ideal work of separation. All the aforementioned studies have defined the qualitatively enhancing or embrittling effect of impurities in Nickel grain boundaries by calculating the segregation energies.

Although a considerable number of elements have been studied theoretically, it is hard to make a comparison and draw conclusions from the available results in the literature due to model differences, impurity concentrations, and calculation approaches basis. Since we are trying to understand the mechanisms and effects of different impurity atoms which involve very small energy differences, it is important to conduct calculations on equal discrepancies for all impurities i.e. using the same model and approximations for each type of targeted physical property calculation. This should allow us to make sensible comparisons and draw meaningful conclusions.

## Models and Computational Details

### Models used in Ni Σ5(210) STGB and (210) surface

**Figure 1:**
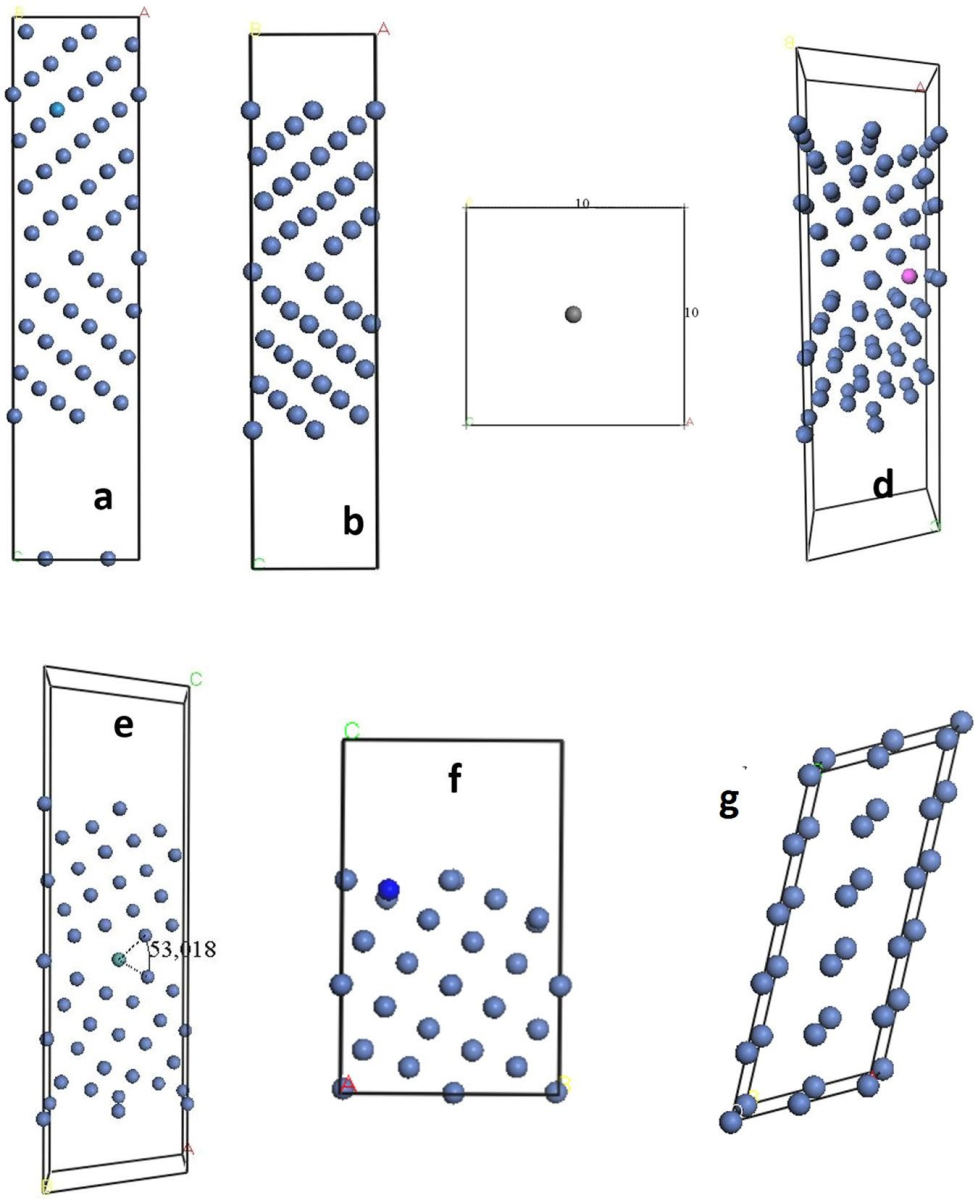
(**a**) Fully relaxed clean grain boundary, **(b)** isolated impurity atom in 1 × 1 nm box to calculate the energy of isolated impurity. **(c)** Fully relaxed clean grain boundary with the impurities deep in the bulk region, **(d)** fully relaxed grain boundary with segregated impurity atoms in interstitial site 0. **(e)** Fully relaxed grain boundary with segregated impurity atoms in substitution site 1. **(f)** Relaxed free surface with segregated impurity atoms. **(g)** Nickel single crystal supercell with the same number of atoms and approximate size used to have the energy of one Ni atom in the bulk.

Face centered cubic FCC Σ5(210) 〈001〉 GB is formed by tilting two symmetric grain by 53.1° with respect to a common 〈001〉 tilt axis with the boundaries plane parallel to (210)^9^. Since we are calculating different properties (segregation energy, surface energy and the tensile strength) for all 19 impurities, we used different models depending on the calculated properties and the required impurity concentration (Fig. 1).

For segregation energy calculations, and due to the remark of Šob *et al*.^10^, which states “*calculations of segregation energies at interfaces are often unreliable when the bulk solid solubility of the segregate is lower than that corresponding to one solute atom per computational repeat cell*. *The calculated energy of a solute in the bulk cannot be used when evaluating segregation energies*”, we used a 2 × 2 unit cell model that generated 80 atoms distributed on 11 layers with P1 symmetry [see Fig. 1(c)]. Thus, the interaction between impurities is negligible. For the calculation of impurity segregation energy to the surface, we also used 2 × 2 Ni unit cell model with 60 atoms distributed over eight layers [see Fig. 1(f)]. The GB unit cell used in tensile strength calculations has 44-atoms, i.e. one impurity per two-repeated GB unit cell [see Fig. 1(a)]. We have to mention that in order to get relevant and comparable total energies, and for each above calculated property for all considered impurities, we used the same calculation parameters, the same GB unit cell size and the same number of atoms per model. This allowed us to calculate the energy difference when the impurity segregates to the surface, bulk and GB for each property. Furthermore, all calculations were carried out with P1 symmetry by forcing symmetry breaking of models, in order to get relevant and comparable total energies

The calculated Ni-lattice constant is 3.518 Å (the experimental value is 3.524 Å). Dimensions of the unit cell of GB model are then 3.5 × 4.3 × 24.5 Å^3^ (Fig. 1). We used 2 × 1 × 1 model for tensile strength calculation and 2 × 2 × 1 cell unit model for segregation energy. The unit cell has a vacuum region, with a width set to 1 nm for segregation calculations and 1.5 nm for tensile strength calculations.

### Computational details

We used pseudopotentials plane wave method for total energy calculations as implemented in Cambridge serial total energy package (CASTEP)^11^ with Norm-conserving pseudopotentials (NCP) and 720 eV cutoff energy for all calculations. Local density approximation LDA CA-PZ was used for the exchange-correlation potentials (CA-PZ: Ceperley–Alder^12^, data as parameterized by Pedrew-Zunger^13^). For the present of GB unit cells, we considered k-points sampling on different models as follow: 4 × 4 × 1 k-points for the 80-atom model and 3 × 5 × 1 for the 44-atom model. These parameters lead to an adequate accuracy of 0.1 meV/Å per atom. A precision of 1 meV was thus sufficient for calculating energy differences. For density of states and population analysis calculations, we used 5·10^−7^ eV as strict criteria for total energy tolerance, together with 6 × 6 × 2 in k-point sampling. Nickel GBs with transition metal impurities poses unique challenges in electronic structure calculations^14^. After several tryouts, norm conserving pseudopotentials and (LSDA) were found to be the best combination that give well converged energies to the required tolerance in all cases and in agreement with the observations reported in ref.^15^.

## Results and Discussion

### Effect of light elements in NiΣ5 GB and its free surface

**Figure 2:**
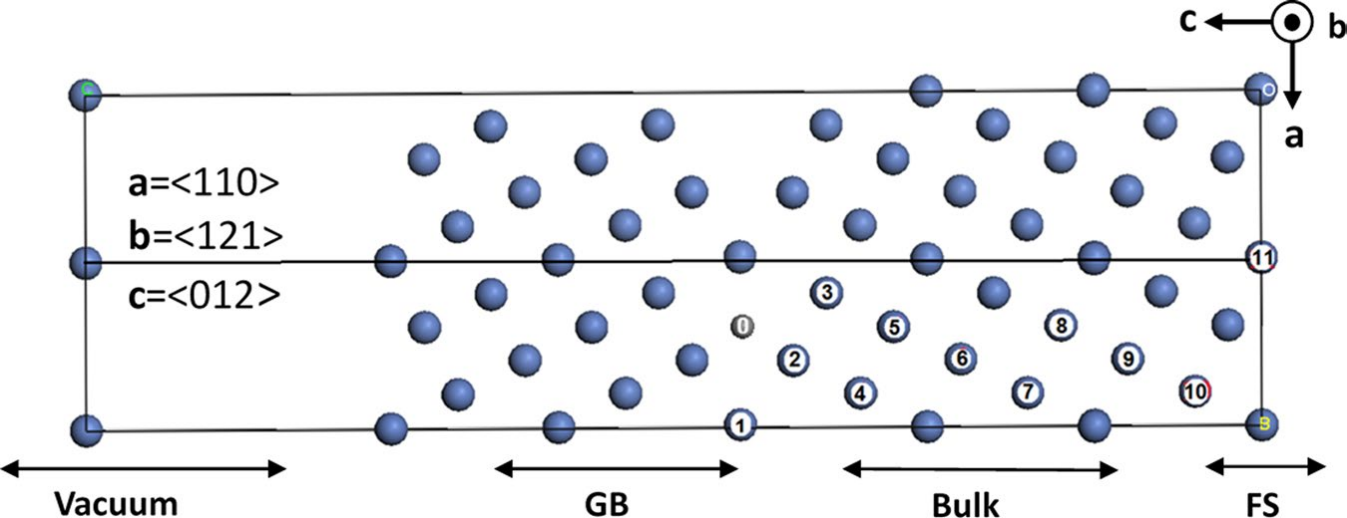
Unit cell model of NiΣ5(210) symmetrical tilt grain boundary, model used in segregation study. Unit cell orientation and direction are indicated. Segregation atomic sites are indicated by numbers from 0 to11. 0 for interstitial.

The calculated segregation energies of the 8 impurities to Ni Σ5 (210) symmetrical tilt grain boundary are presented in Table 1. These energy values correspond to an energetically minimum position. These positions are picked up from the segregation energy calculations in different possible sites in surface (sites 9, 10, 11) and in the GB region (sites 0, 1, 2, 3) (see Fig. 2). Some of the minimal positions used, especially in GB region, were extracted from the literature. For example, Yamaguchi *et al*.^16^ have reported that site 2 is the most stable for sulfur system. Results published in refs^2,17^. have predicted an interstitial segregation of all six nonmetallic elements and substitutional for Aluminum. In the present work, we used site N°7 to calculate the binding energy of an impurity in the bulk, and make it as reference in segregation calculation. For each configuration, we performed structural relaxation of the impurities atoms, mainly 5 to 6 close neighboring Ni atoms while fixing the rest of the Ni atoms of the system.

**Table 1:** The Binding energy E_b_ of 8 light impurities in the Bulk and in the most stable Ni Σ5GB.

Formation energies						Segregation energies					
	Bulk	GB	other	FS	other	GB	other	Surf	other	RWEP	Other
B	−5.94	−8.82	−7.14	−7.08	−6.30	−2.08	…	−1.14	…	−0.94	−0.84
S	−4.40	−6.63	−4.72	−6.01	−5.78	−1.77	−1.4*	−2.67	−2.32*	0.892	1.06/0.99*
C	−7.23	−9.82	−8.2	−9.29	−7.87	−1.71	…	−2.06	…	0.34	−0.33
O	−4.10	−6.79	−5.79	−7.27	−6.66	−2.25	…	−3.17	…	0.91	0.87
N	−4.79	−8.29	−7.5	−7.74	−7.85	−2.83	…	−2.95	…	0.12	0.35
P	−3.89	−6.18	−6.45	−5.71	−6.44	−1.72	−1.6*	−1.82	−1.53*	0.10	−0.01/−0.07*
Al	−5.51	−5.78	…	−5.61	…	0.05	−0.22*	−0.10	−0.19*	0.16	−0.30
Si	−7.44	−8.63	…	−7.67	…	−0.71	−0.76	−0.39	−0.35	−0.32	−0.41

In the right side the Segregation Energy E_seg_ of impurities to the grain boundary and to the surface together with the Rice Wang embrittling potency. The comparison with previous works is shown.

*Data taken from^2^, other: data are taken from^17^.

The binding impurity energies $\Delta {E}_{b}^{GB}$ to the grain boundary in both substitutional and interstitial segregation positions are reported in Table 1. Our results are in good agreement with literature data. According to the Rice–Wang model, large negative value of the binding energy of the impurity at the GB means stronger bonding between the impurity and nickel atoms^18^. Light elements have high segregation energies to the GB especially N by −2.8 eV, and higher segregation to the surface was observed for S and Si elements. However, phosphorus shows a weak segregation to the FS and GB. Boron has the highest enhancing potency of 2.84 eV, which is according to the Rice-Wang embrittling potency (RWEP) considered as the best enhancer^18^.

**Figure 3:**
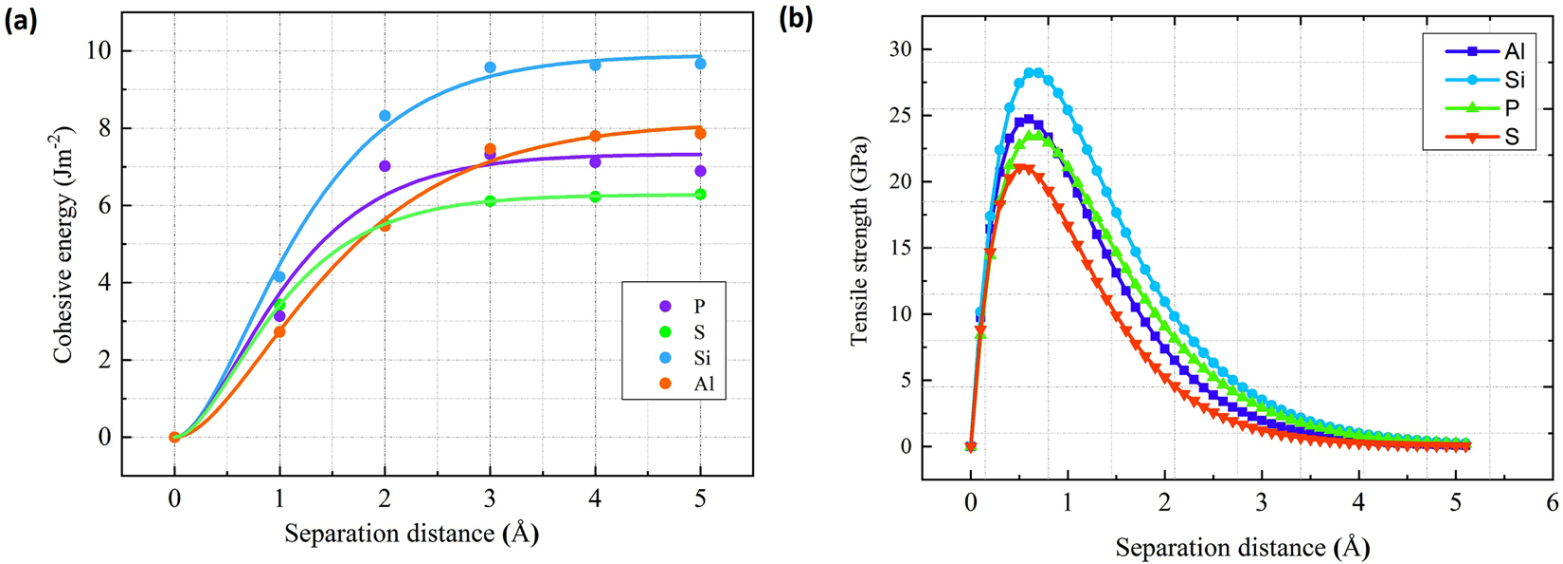
(**a**) Cohesive energy in J.m^−2^ and **(b**) tensile stress in GPa of the NiΣ5(210)GB as a function of the separation distance for Al, Si, P and S. The interlayer distances of a perfect bulk in « c » direction is taken as a reference for separations in all configurations.

In order to fully investigate the effect of impurities segregation in Ni GB, we need to further calculate two fundamental values: the cohesive energy (E_coh_) and the theoretical tensile strength (TTS). As mentioned in Tahir and Janisch work^18^, cohesive energy (from *ab-initio* calculations) alone is not sufficient to describe fracture processes. In the present work, E_coh_ and TTS were calculated for 8 light elements (N, B, C, Al, Si, P, O and S), with the 44 atoms model. Results are gathered in Table S1 (Supplementary Information). It is important to mention that in all considered cases the interlayer separation distance is parallel to the *z*-direction of the GB model (Fig. 2). Figure 3(a) shows the evolution of the calculated cohesive energies as a function of the separation distance between the two grains in the tensile process fitted to the Rose universal binding curve for Al, Si, P, S. Figure 3(b) shows the TTS change versus the separation distance between the two grains. The maximum tensile strength is located around 0.5 A.

### Effect of transition metal elements in NiΣ5 GB and surface

The purpose of the present sub-section, is to present a systematic *ab-initio* study the transition metal elements effects belonging to 4^th^ to 7^th^ columns of the periodic table, namely: Tc, Ti, Cr, Zr, Nb, Mo, Mn, Hf, Ta, W, V and Re, on Ni Σ5 (210) tilt GB and surface. For these impurities, we calculated segregation energy, strengthening/embrittling energy, cohesive energy, binding energy and the tensile strength. Based on these identification factors and by comparing our results with data from the literature we aim to conclude this section with a clear answer on the effect of these impurities on Nickel GB. Table 2 reports the calculated binding as well as segregation energies to the surface and to the Ni Σ5 (210) GB for the considered 11 transition metal impurities. It is well known that the impurity segregates to energetically minimum position. This stable position is identified from the results of segregation energy calculations in different possible sites: in surface sites 9, 10, 11, and in the GB region: sites 0, 1, 2, 3 (Fig. 2). Here Again, some of the minimal position sites, especially those of the GB region, are extracted from the previous works in literature. Razumovskiy *et al*.^6^ found that site1 is more stable than the interstitial site 0 for all the transition metals cases he considered in in study. This is obviously in good agreement with our present results. From the detailed study of vanadium impurities^15^, we concluded that site 1 is the most stable (Fig. 2), which is also confirmed by previous studies especially by Yamaguchi *et al*.^9^, who considered site 1 for transition metal elements. On the other hand, results presented in Table 2 show that Zr, Hf and Ta have the strongest binding energies, while Mo, V, Cr have the weakest one. The values of Mn binding energies to the GB and FS show an embrittlement effect. In the next section, we will report a detailed electronic structure study of the effect of Mn in order to explain this situation. A first remark that arises from Table 2, is that Zr has the highest GB segregation energy, but weak RWEP. This situation corroborate the well-known role of Zr in Nickel based alloys^19^. Moreover, Nb, Re, W have also high segregation energies to the GB with large RWEP, which makes them potential candidates to play a role as strengthening agents to Ni GB. These results are in good agreement with other works^5,7,20^. It is worth mentioning that segregation energy of Mo and Ti are calculated for the first time, and hence can be considered as theoretical predictions. Furthermore, Ti has an acceptable embrittling potency (−0.34 eV) which leads to remarkable tensile strength (see sec.III.4). Cr and Mo have large positive segregation energies. Simultaneously, they present good TTS and reasonable cohesive energy. This leads to consider the value of segregation energy as doubted since the nonmagnetic results found by Young *et al*.^21^ have shown more acceptable segregation energy results. Among the considered 11 transition elements, Mn shows an abnormal behavior as it has large RWEP.

**Table 2:** The Binding energy E_b_ of 12 transition metals impurities in the Bulk and in the most stable NiΣ5 GB site (generally site 1).

Binding energies (eV)				Segregation energies (eV)					
	Bulk	GB	FS	GB	other	Surf	other	RWEP	other
Ti	−7.83	−7.99	−7.65	−0.16	…	0.18	…	−0.34	…
V	−3.48	−4.1	−3.86	−0.62	…	−0.38	…	−0.24	…
Cr	−3.43	−1.04	−4.25	1.7	−0.02^b^	−0.54	−0.61^2^	2.24	−0.53^b^
Mn	−3.02	0.21	−0.20	3.2	+0.19^b^	2.8	+0.39^2^	2.7	−0.2^2^
Zr	−7.88	−9.10	−8.59	−1.22	−1.94^a^–1.02^b^	−0.72	−1.77^a^–1.01^b^	−0.51	−0.17^a^ + 0.08^b^
Nb	−5.38	−6.14	−5.52	−0.76	−0.49^b^	−0.14	−0.03^b^	−0.62	−0.46^b^
Mo	−3.18	−3.21	−3.21	−0.45	…	−0.2	…	−0.25	…
Hf	−8.51	−8.68	−8.68	−0.78	−1.65^a^	−0.17	−1.16^a^	−0.61	−0.49^a^–0.8^c^
Ta	−9.02	−8.68	−8.68	−0.57	−0.93^a^	0.34	0.13^a^	−0.91	−1.05^a^
W	−6.50	−6.05	−6.05	−0.13	−0.45^a^	0.45	0.87^a^	−0.57	−1.32^a^
Re	−5.72	−5.25	−5.25	−0.11	−0.12^a^	0.47	1.21^a^	−0.58	−1.33^a^

In the right side the Segregation Energy E_seg_ of impurities to the grain boundary and to the surface together with the Rice Wang embrittling potency. The comparison with previous works is shown.

^a^data taken from^5^, ^b^data taken from^21^, ^c^data taken from^7,23^.

**Figure 4:**
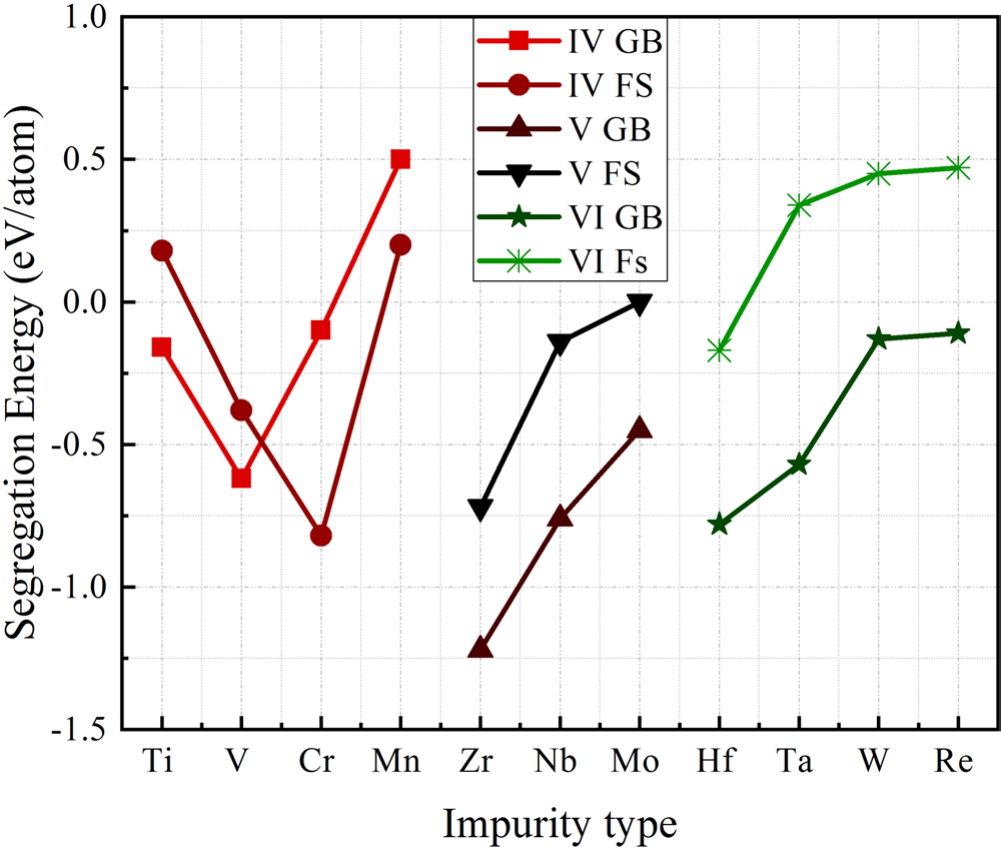
Segregation energies to the GB and surface calculated for the considered 11 transition metal impurities, the order is baed on the periodic table lines.

Figure 4 depicts the impurity segregation energy to both GB and FS as function of impurity type arranged according to increasing atomic number and period in the periodic table of elements. RWEP values are changing considerably as function of the period (Table 2). The more we move away from Ni in period 4, the higher the RWEP is. We can also see that there is no linear relation between the size of impurity and segregation energy, in contrast to the light elements case.

**Figure 5:**
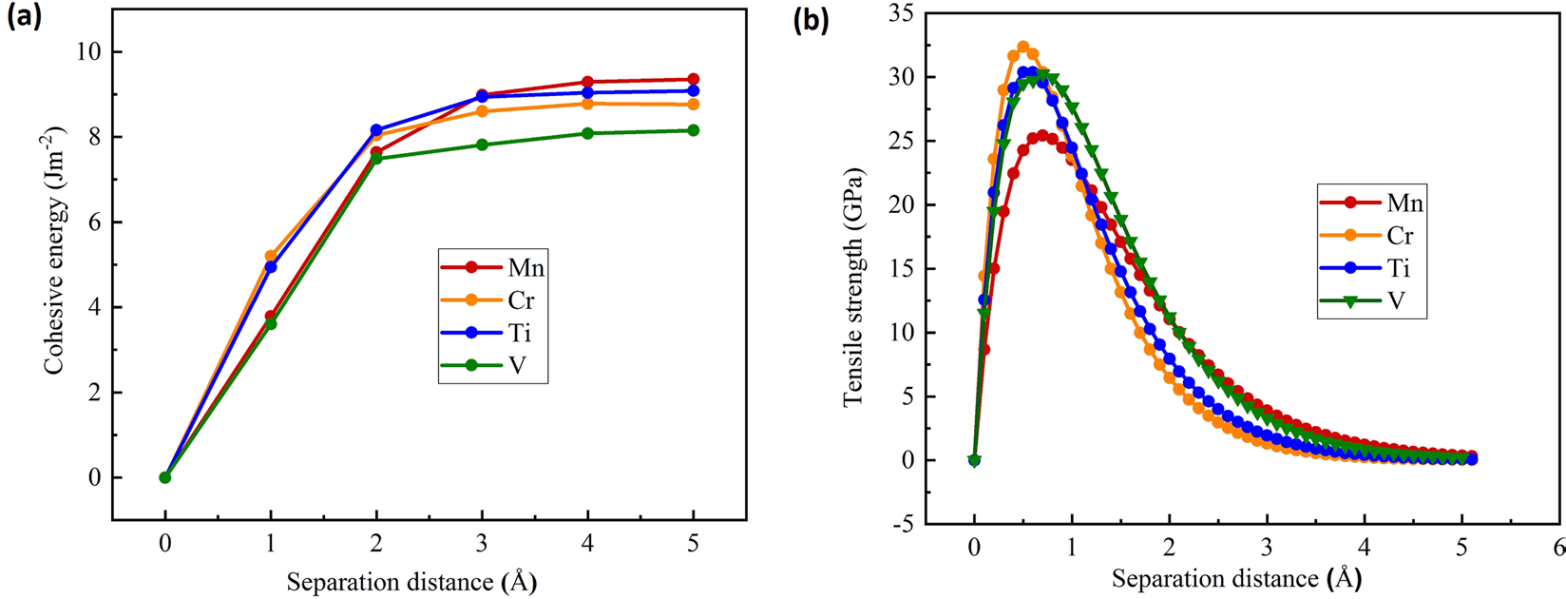
(**a**) Cohesive energy in J·m^−2^ and (**b**) tensile stress in GPa of the Ni Σ5(210)GB as a function of the separation distance for the period 4: Ti, V, Cr and Mn.

We further present here results and discussion of two fundamental properties: cohesive energy and theoretical tensile strength (TTS), calculated for (Ti, V, Cr, Mn, Zr, Nb, Mo, Tc, Hf, Ta, W, Re, Tc) impurities in Nickel GB in substitution site1, as summarized in Table S1. Figure 5(a) shows results for evolution of the cohesive energies, fitted to Rose universal binding curve, as a function of the separation distance for 4 impurities of the 4^th^ row of the periodic table namely Ti, V, Cr, Mn. Figure 5(b), displays the calculated TSS for the considered transition metal impurities. It is evident to see that Mn and Mo elements are not good enhancers. In fact, Mn presents embrittling effect, while Mo has no effect on the TTS although it increases the cohesive energy of the grain boundary to 4.71 eV (Table S2). With close segregation energies, RWEPs for Mo, Ti and Mn are −0.42, −0.34 and +3.03 respectively. So, from the segregation point of view the behavior is the same, but their mechanical effect on Ni GB is quite different. Mo has approximately no effect on the GB with 27 GPa, whereas Ti is an enhancer with 31 GPa, and Mn is the only embrittling transition metal to the GB with 25 GPa (Table S2). Further explanation of Mn effect using electron density difference will be discussed in the following section.

**Figure 6:**
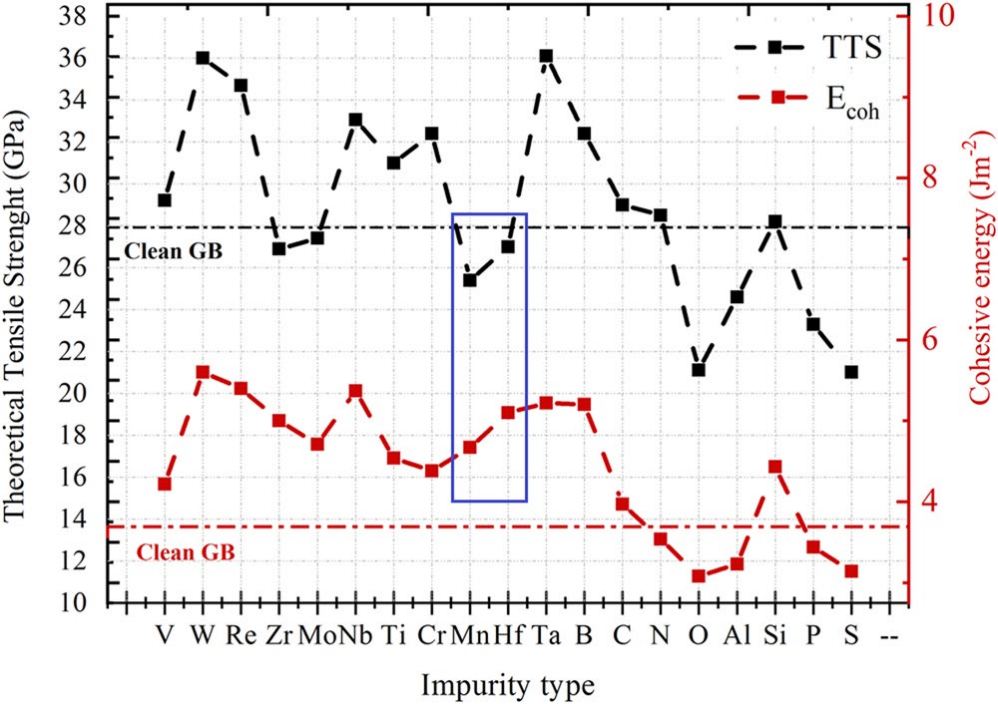
(red) the value of cohesive energy in Jm^−2^ with function of impurity type in Ni ∑5(210) grain boundary. (black) the values of the calculated tensile strength for the same conditions. Impurity concentration is at 0.5 atm/ML. The blue triangle refer to a case when cohesive energy and tensile strength disagree in defining the enhancing/embrittling effect.

To summarize, Fig. 6 groups calculated values of cohesive energy together with tensile strength of the considered 19 elements in one plot. Although a correlation between the TTS and cohesive energy seems to appear for most of the elements, cases like Mn, Hf and Zr show an enhancement of cohesive energy with weakening in TSS, which confirm the results of Tahir *et al*.^18^. More discussion about the impurities effect separately can be found in ref.^19^.

### How B, S, V, Nb, Mn and W affect nickel ∑5 grain boundary?

Segregation energy and tensile strength calculations can provide us with an insight on the enhancing and the embrittling effects of impurities in the GB, but they cannot provide us with the answer of the question: why? The scrutiny of the electronic structure based on density of states, electron density difference and population analysis can help us to answer why an impurity is an enhancer or embitterer. Presently, we study the effect of Tungsten and Manganese as they were found the most enhancing/embrittling impurities for our metallic case. The choice is based on their different influence on Ni GB. From section III.2, we found that Tungsten is an excellent enhancer, and Manganese is an embrittler. Both of them form a metallic bonding. The question is: why we found a considerable difference in cohesive energy that made Tungsten strong enhancer and Manganese embrittler?

**Figure 7:**
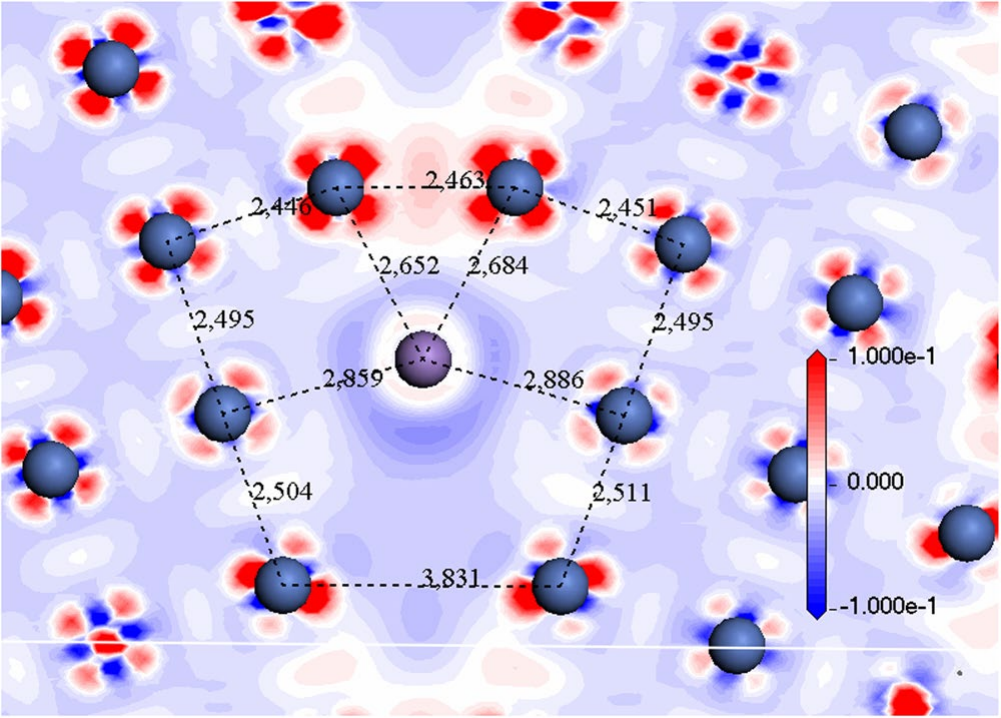
The charge density difference (in electrons/Å^3^) of the Ni∑5(210) GB with substitutional segregated Manganese impurity (in purple) in site 1. Contours start from 0.1 to −0.1 e/a.u^3^. Distances in Å.

**Figure 8:**
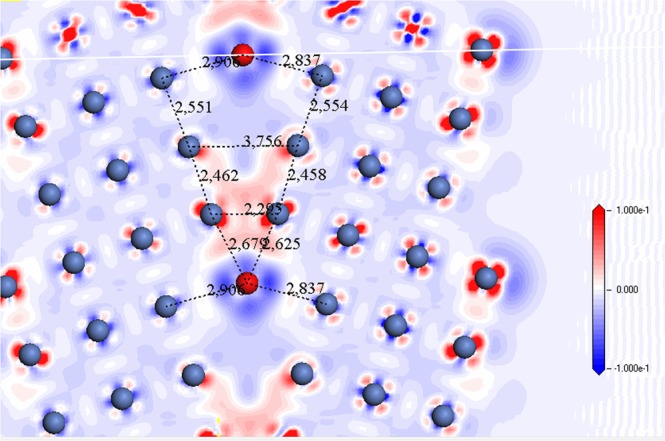
The charge density difference (in electrons/Å3) of the Ni∑5 GB with substitutional segregated Tungsten impurity (in red) in site 1. Contours start from 0.1 to −0.1e/a.u^3^. Distances are in Å.

On one hand and from a structural point of view, it is clear from Table S2 and Fig. 5, that Mn caused to expansion of GB and enlarges the distance between site 3 and its symmetrical position site 3′ which expands from 2.53 Å for pure Ni GB to 2.83 Å when Mn has impurity in site 1, Fig. 7. On the other hand, Tungsten results in only 2.54 Å for Ni2-W and 2.7 Å for Ni4-W and form a 2.75 Å bond length between Ni3-Ni3′ (Fig. 8). We observe that tungsten did not cause much expansion of GB even though its atomic radius is larger than Mn’s (193 pm for W and 161 pm for Mn). This effect is not related to bond type because both of them are metal, nor to the size of impurity atom. From the electron density difference, we can see strong perturbation of the charge around tungsten, which is not the case for Mn and that means a weaker bond.

**Figure 9:**
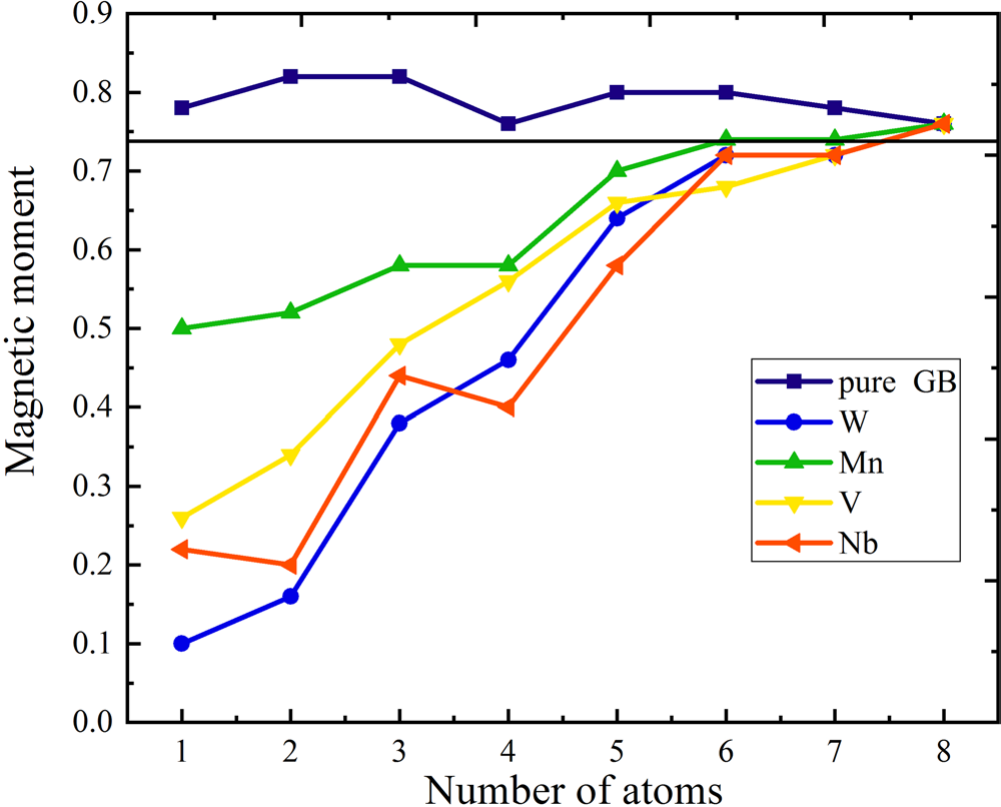
The magnetic moment values of Ni atoms labeled by sites number at the NiΣ5 (210) GB with substitutional segregated W, Mn, V, Nb and clean GB. The line at 0.74 µ_b_ represent the calculated value of magnetic moment of Ni crystal. Number of atoms are indicated in Fig. 1.

To further investigate the properties of GB, we analyzed the magnetic moment of two cases in order to elucidate its effect on Ni GB properties^20,21^ as displayed in Fig. 9. Tungsten induces a strong reduction of the magnetic moment of nickel atoms around it in the GB and generates magnetically dead layers as shown in Fig. 9. This is not the case for Mn that produces the weakest perturbation of Ni magnetic moment. Moreover, we noticed a different behavior as far as the change of the magnetic moment for the isolated atom (m_Mn_ = 5 μ_B_, m_W_ = 3 μ_B_) and its magnetic moment in the GB (m_Mn_ = 2.31 μ_B_, m_W_ = −0.03 μ_B_). The change in magnetic moment of impurities in the GB equal to m_Mn_ = 5.2 μ_B_ and m_W_ = 0.06 μ_B_. The value of the magnetic moment for the pure Nickel single crystal calculated with population analyses in CASTEP was found to be 0.74 μ_B_. This value is larger than the experimental one (i.e. 0.61 μ_B_) and close to previous DFT studies^22^. These high values of magnetic moment are also in the GB models compared with previous works, but they reproduce the same shifted results with the order of atoms position in Ni GB model (generally higher by 0.12 μ_B_). We consider that the main reason for this shift is due to the Norm-conserving pseudopotentials, as they are known to overestimate the magnetic moment^14^. It should be noted that because we are doing a systematic and comparative study, all impurities effects were calculated under the same conditions, therefore our results of magnetic moment should be taken for comparison purpose only. The presence of impurities in the substitutional position induces the creation of magnetically dead layer in the GB region with different amounts (Fig. 9). Site N°7 was considered in segregation energy as the reference point for the bulk. It is also valid as a magnetic reference for entering the bulk region. As we can see here, the value of its magnetic moment corresponds to that of pure Ni single crystal (0.74 µ_B_). After that in site N°8, all points meet together in the same value which means that the magnetic impurity effect on the Ni GB model is vanishing.

**Figure 10:**
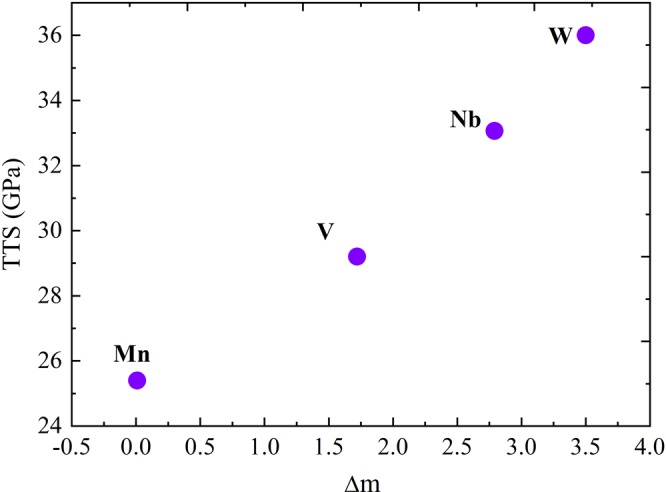
Theoretical tensile strength TTS (in GPa) versus magnetic moment difference between isolated atom and atom in Ni GB Δm (in magneto Bohr, μ_B_).

To get some trends from the obtained results, we compared the loss of magnetic moment in the GB region and the TTS for Mn, W, V and Nb. We define Δm as the difference between the magnetic moment of impurity in the GB and when isolated. We plotted Δm versus TTS in Fig. 10. We see clearly the larger Δm is, TTS increases. Tungsten has the highest Δm followed by, Nb, V and then Mn (Fig. 10). This trend is not applicable for non-metal impurities (B and S) because they are non-magnetic elements. It should be noted that Δm has a positive value for the enhancing element W, Nb, V. It has a negative value for weakening element (Manganese), because Mn atom gains a magnetic moment of 0.04 μ_B_ thus Δm = −0.04 μ_B_. This important result lead us to directly relate the sign of Δm to the strengthen/embrittling effect. This sign trend is also valid even for the non-metals elements, such as sulfur (Δm = −0.06 μ_B_) and boron (Δm = +0.08 μ_B_). This approach differs from the approach of Tian et *al*.^22^. In fact, in their work, they were interested in the magnetic moment of the bulk and found it to reduce much of the energy release upon fracture, and not the variation of magnetic moment of the impurity itself.

## Conclusion

A systematic study on the effect of combination of light and transition metal elements in grain boundary has allowed us to find and discuss some trends between the cohesive energy and TTS. The comparison of our results conducted on the effect on Ni GB of eight light elements to those in the literature (theoretical and experimental data) have shown a good agreement. Our main contribution was to determine, the cohesive energy of: O, Si, Al, N in Ni∑5 (210) GB and the tensile strength values which was uncovered topic for C, O, Al, Si, N in Ni∑5 (210) GB. Furthermore, we explored new insights to predict if an impurity is an enhancer or embitter. Our major finding is based on the loss or gain of magnetic moment (and not the atoms in the matrix) of the impurity, as an isolated atom and when in the GB. The higher the loss, the more the impurities consolidate the GB. The gain in the magnetic moment for the impurity causes embrittlement. Further investigations are recommended to check out the validity of the theoretical approach on different magnetic systems. A study about the segregation energy variation and tensile strength with function of impurities electronegativity, and co-segregation of the most enhancer element (Tungsten) and the most embrittler (Sulfur) in NiΣ5 grain boundary is presented in the Supplementary Materials.

## Supplementary information

supplementary information
