# Supplementary material for: Toward a better understanding of the enhancing/embrittling effects of impurities in Nickel grain boundaries
Source: Sci Rep. 2019 Oct 1;9:14024. doi: 10.1038/s41598-019-50361-3 (PMC6773772; doi:10.1038/s41598-019-50361-3)
Supplement: Supplementary file 1 — supplementary information [file 41598_2019_50361_MOESM1_ESM.docx]

**Toward a better understanding of the enhancing/embrittling effects of impurities in Nickel grain boundaries**

EL Tayeb Bentriaa, Ibn Khaldoun Lefkair^a^, Ali Benghia^a^, Bachir Bentria^a^, Mohammed Benali Kanoun^b^ and Souraya Goumri-Said^c^

*^a^Laboratoire Physique des matériaux, Université Ammar Telidji de Laghouat; BP 37G Laghouat 03000, Algeria*

*^b^Physics Department, College of Science, King Faisal University, P.O. Box 400, Al-Ahsa* 31982*, Saudi Arabia*

*^c^College of Science, Physics department, Alfaisal University, P.O. Box 50927, Riyadh 11533, Saudi Arabia*

**(Supplementary information)**

In the present work, E_coh_ and TTS were calculated for 8 light elements (N, B, C, Al, Si, P, O and S), with 44 atom model. They are listed in table S1.

**TABLE S1.** Cohesive energy (in J·m^−2^), tensile strength (GPa) of NiΣ5(210) grain boundary and 8 light impurities in site 1.

|  | **E_coh_(eV)** | **TTS(GPa)** |  | **E_coh_(eV)** | **TTS(GPa)** |
| --- | --- | --- | --- | --- | --- |
| **Pure GB** | 3.65 | **27.60** |  |  |  |
| **B** | 5.2 | **32.4** | **Al** | 3.23 | **24.6** |
| **C** | 3.97 | **29** | **Si** | 4.43 | **28.2** |
| **N** | 3.54 | **28.5** | **P** | 3.44 | **23.3** |
| **O** | 3.08 | **21.1** | **S** | 3.14 | **21.0** |

|  | | | | | |
| --- | --- | --- | --- | --- | --- |
|  | cohesive | TTS | relaxed | cohesive | TTS |
| **V** | 4.22 | 29.2 | Ti | 4.54 | 31.0 |
| W | 5.60 | 36.0 | Cr | 4.38 | 32.4 |
| Re | 5.40 | 34.7 | Mn | 4.67 | 25.4 |
| Zr | 5.00 | 26.9 | Hf | 5.10 | 27.0 |
| Mo | 4.71 | 27.4 | Ta | 5.22 | 36.1 |
| Nb | 5.37 | 33.06 |  |  |  |

**TABLE S 2.** The cohesive energy (in J·m^2^) and corresponded theoretical tensile strength (in GPa) for pure Ni∑5(210) GB and with different transition metal impurity types.

**Variation of segregation energy and tensile strength with function of impurities electronegativity**

The variation of segregation energy with the function of electronegativity is presented in Fig. S1. There is a correlation between the electronegativity of impurities and segregation energy especially to the surface. The stronger the electronegativity, the higher segregation energy are to the GB/surface ratio. For example, Oxygen and Nitrogen have the highest electronegativity with 3.5 and 3.0 respectively and they show the highest segregation energy to the surface and GB. Aluminum has the weakest electronegativity (equal to 1.5) and shows the lowest segregation energy. This trend is not applicable in transition metals (as discussed in section III.2). Moreover, for the considered light elements, our calculation shows that the GB/surface segregation energy has a trend with the atomic number of impurities Fig. S2. Two separated lines appear in Fig. S2, which corresponds to the two colons of periodic table. There is a strong correlation between the size of the impurities and the segregation energy. The 2*p* elements B, C, N, O with atomic ratio between 0.73Å to 0.82Å are much smaller than 3*p* elements Al, Si, P, S with atomic ratio varying from 1.02Å to 1.18 Å. This effect is known in literature[1], and represent the atoms size affecting segregation phenomena in GB [[1](#_ENREF_21)].

As in case of atom size effect and electronegativity of impurities, we have investigated the possibility to find a similar trend between them and the theoretical tensile strength (TTS). Fig. S2, shows a dispersion of results, which means no general correlation. Nevertheless, we remark a correlation between the electronegativity and TTS for every single period of the periodic table as seen in Fig. S2. So the effect of atom size and electronegativities of impurities are dominant in determining the segregation energy, but both of them fail to give a correlation to the strengthen/embrittling effect.

The variation of segregation energy as function of electronegativity in Pauling units is presented in Fig. S3. We saw in section III.1 that there is a strong correlation between electro-negativities of impurities and segregation energy in the light elements. This relation is not true for transition metal elements and there is a large disparity of values available in the literature. So, the remark made earlier in this paper (section III.1) and elsewhere [[2](#_ENREF_2)]“the higher the electronegativity, the stronger the segregation energy” is not valid for transition metals (Fig. S3).


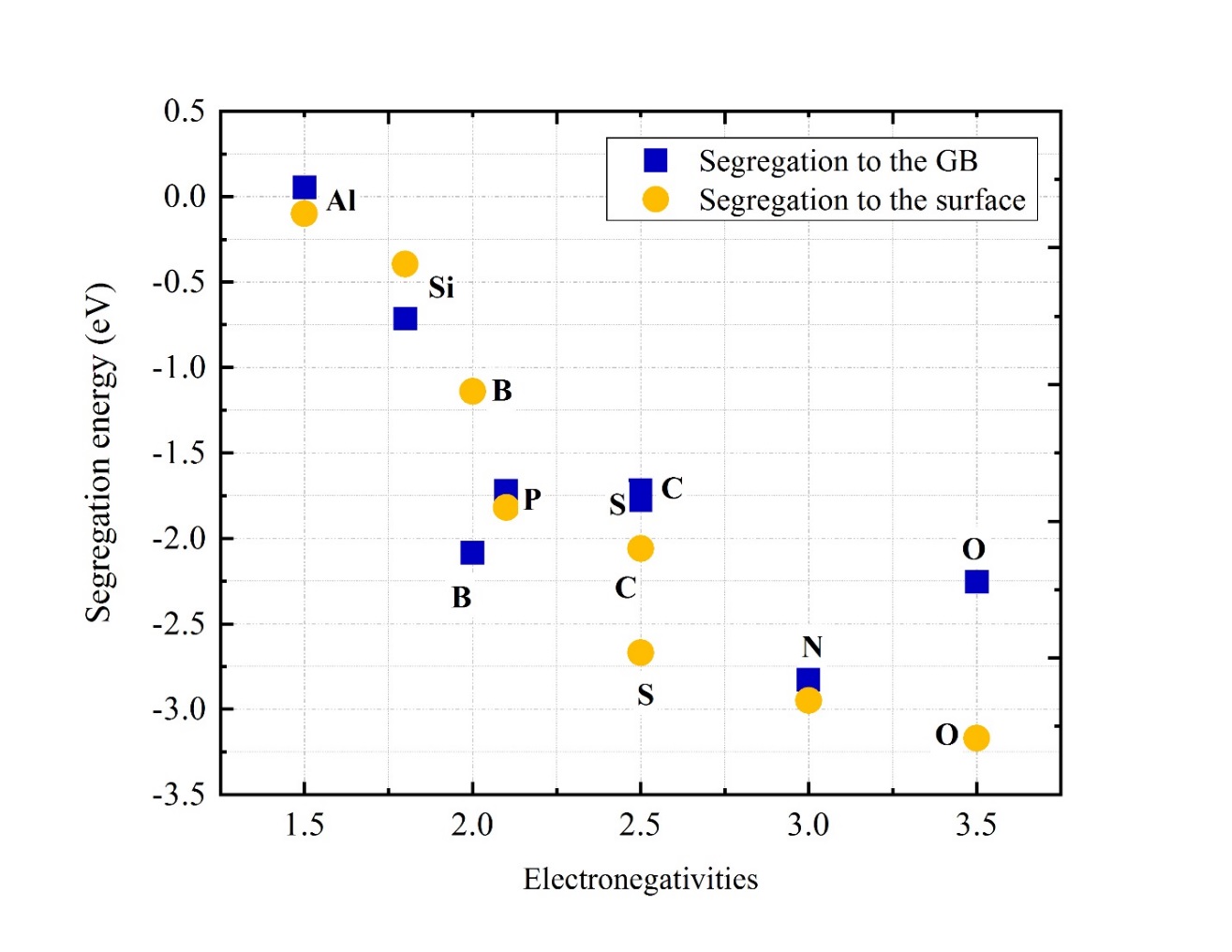


**FIG. S1.** Variation of segregation energy with function of impurity electronegativity for 8 light elements, segregation energy in eV and electronegativity in Pauling units.


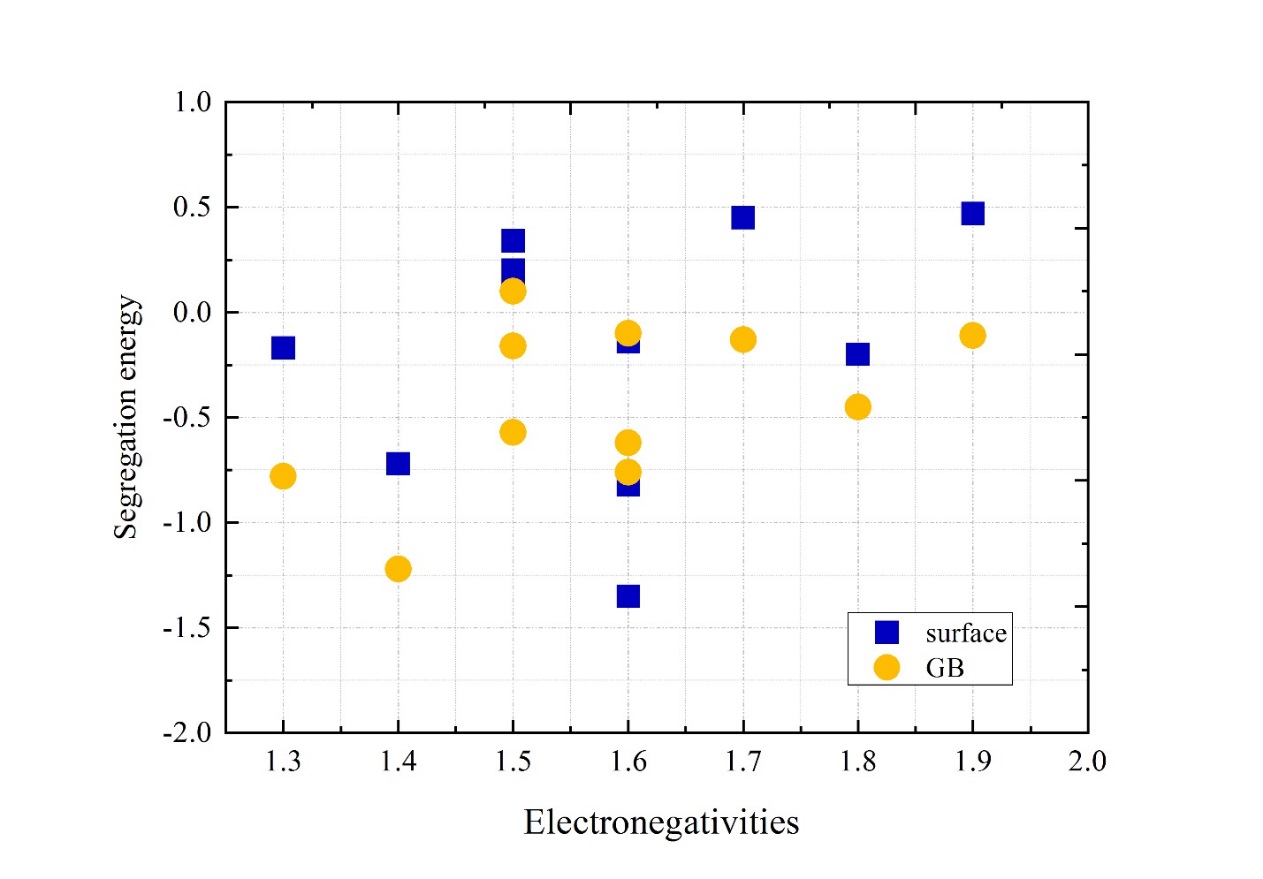


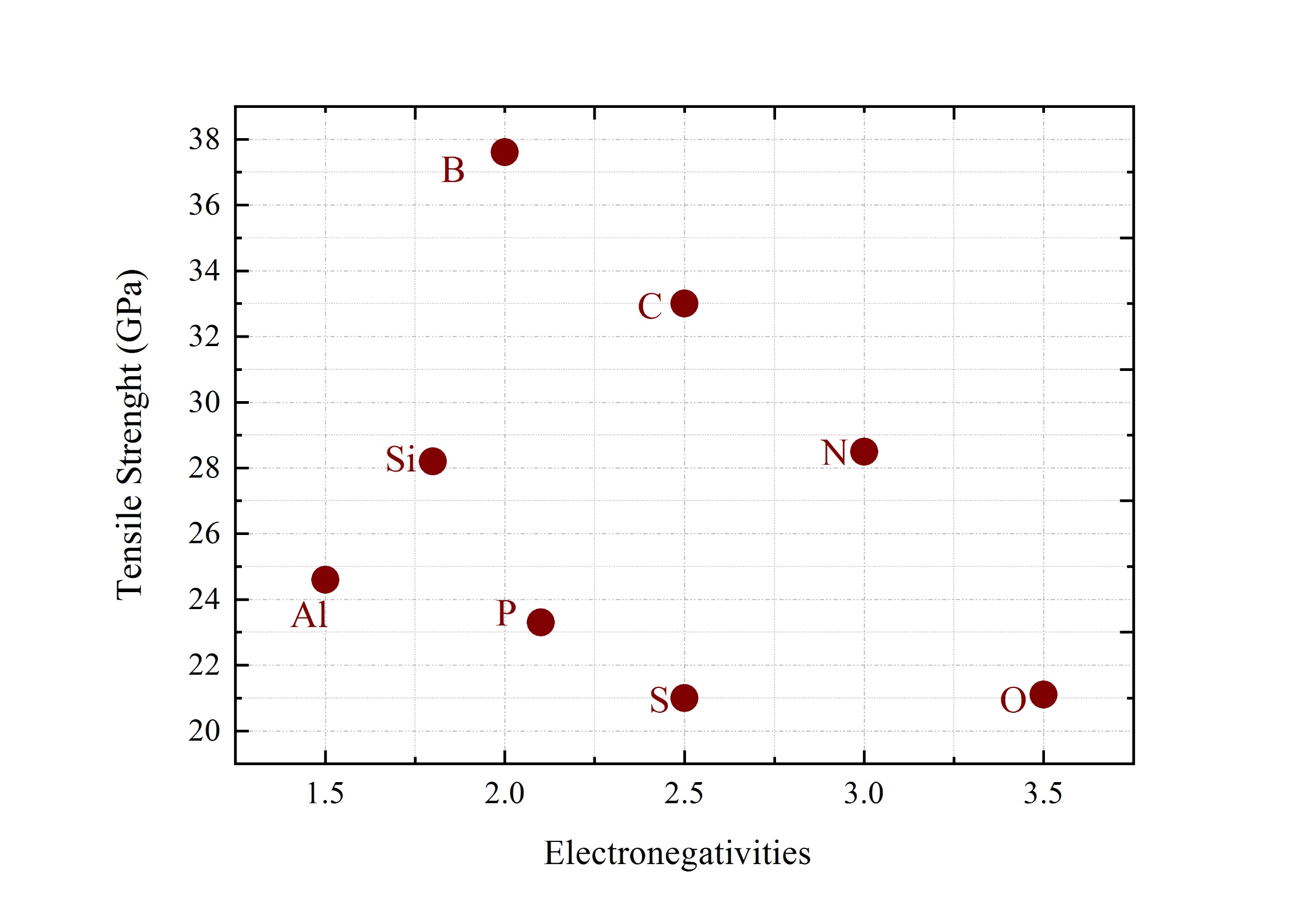


**FIG. S2.** The variation of Tensile strength with function of electronegativity in Pauling units, we can see that both C and S have value of electronegativity equal to 2.5 but they have completely different TTS value.

**FIG. S3.** The variation of segregation energy with function of impurity type for 11 transitions metals, segregation energy in eV and electronegativity in Pauling units

**Co-segregation of Tungsten and Sulfur in Ni Σ5 grain boundary**

We concluded from section III.2 that Re, W and Ta have the highest values of cohesive energy and tensile strength with privilege to W. Form the Rice-Wang Embrittling Potency (RWEP) values the three elements in period 6 of periodic table produce a high tensile strength and cohesive energy in Ni GB. Other theoretical finding shows that W has the highest RWEP [3]. From our selection of 12 impurity transition metals, we choose tungsten as the most enhancer impurity in the Ni Σ5(210) tilt grain boundary and thus we study it as a potential co-segregation enhancer in the case of its presence in GB with sulfur which is shown as the most embrittler element (section III.1).

Eleven models were prepared in order to simulate grain boundary behavior due to co-segregation of tungsten and sulfur atoms. Both experiment and theoretical calculation have reported that the segregation energy of sulfur is still high for large concentrations of sulfur at Nickel grain boundary [4]. In these models we evaluated the concentration of one S impurity for 0.5, 1.0 and 2.0 atom per monolayer (at/ML), and 0.25, 0.5 and 1 at/ML for W impurity in Ni GB. For the co-segregation case, we studied five possibilities, 2S1W which means 2.0 at/ML of S and 1.0 at/ML for W. 1S1W with W in two sites 1 and 3, 1S2W and 1S4W (for these sites, see figure 2). We used in the distribution of impurities in the GB region in which sites correspond to the lowest calculated or available results of segregation energy. From [4], sulfur is known to have a habit to segregate at the grain boundary in site N°2 with -1.6eV then to site N°0 with -1.5eV. So, we put one S atom in site 1 for 0.5 ML concentration, and two S atoms in site 0 and 1 for the 1ML concentration.

For the cases of one impurity type in Ni GB, the TTS of sulfur is in excellent agreement with [4] work, and for the GB with tungsten, the available results of cohesive energy corroborate with results if ref. [3].


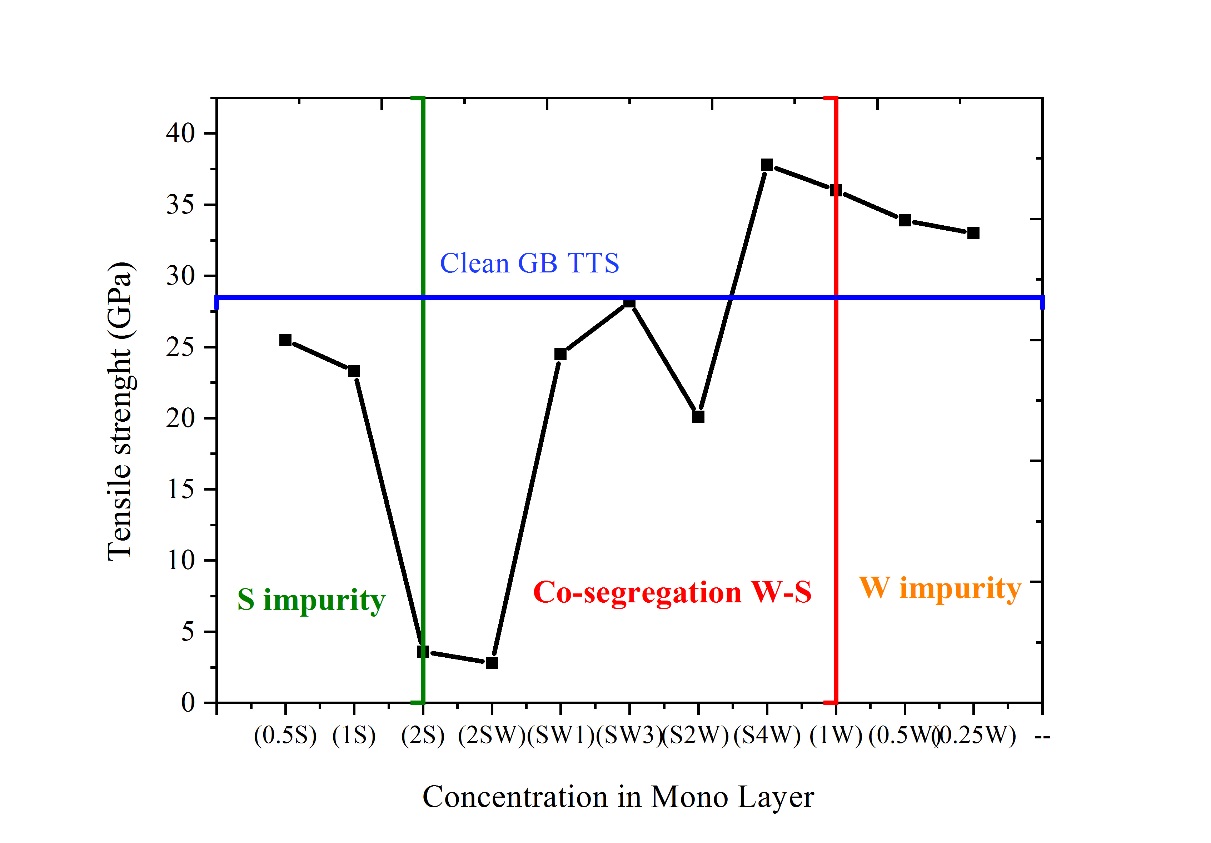


**Figure S4:** Calculated maximum tensile stress (tensile strength) for different concentration of Sulfur and Tungsten, and the strength result from the Co-segregation of S and W with different possible concentrations.SW3 means one S atom and one W atom in site N°3.

For the co-segregation, we might observe that tungsten has none stable enhancing effect, and it depends on atoms concentration and/or position. For example, the presence of tungsten with high concentration of sulfur does not prevent the destructive effect of sulfur. Nevertheless, the increase of tungsten concentration as substitutional sites in the GB region increases the TTS, until S-4W that has a very high TTS. That’s means that only a high concentration of tungsten comparing to sulfur in the GB region could overcome the undesired effect of S and enhance the GB. Also, we note that this conclusion is not applied for the case of 1S-2W that shows an embrittling behavior comparing with 1S only. This behavior is related to the segregation position type that creates much space than the clean Ni GB (site 0 is not occupied in this case).

To understand more the enhancing effect of tungsten, we presented the population analysis performed with CASTEP [5, 6] for the particular case of S-2W concentration (Tables S3 and S4). Figure S5 displays electron density difference in 3D isosurface. It can be seen that the GB region, responsible on the fracture behavior, have a clear loose of charge. The position of sulfur between the two tungsten atoms repulses them out of the GB vacancy site and create larger space than the pure Ni GB system.


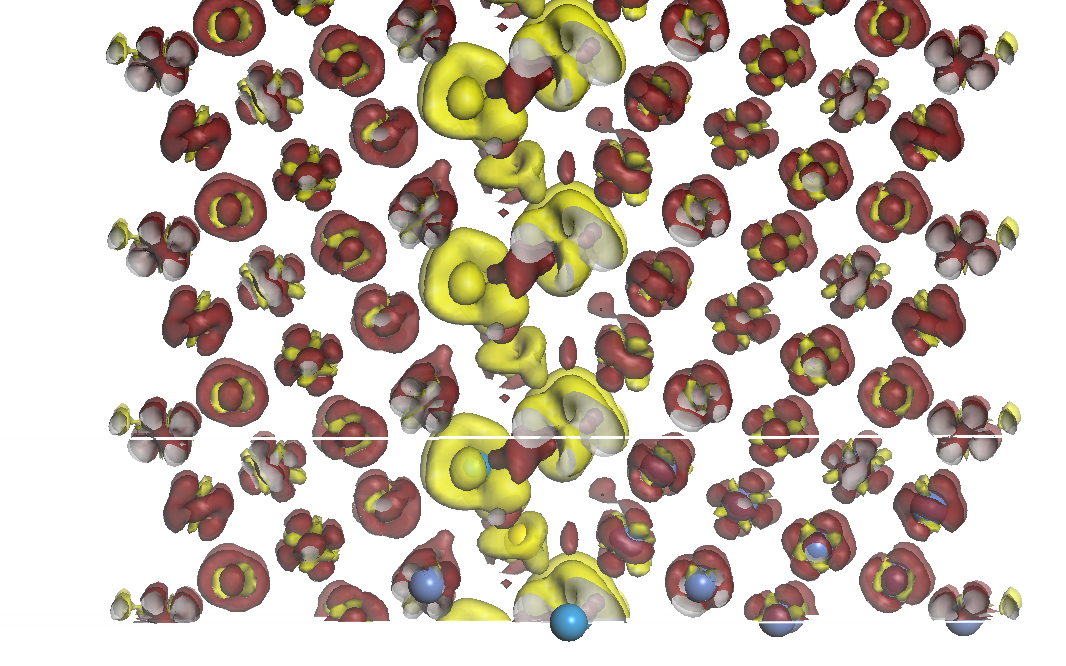


**S**

**W**

**Figure S5:** The 3D electron density difference of concentration S-2W. The yellow isosurfaces mean loose of charge. The two green lines represent the asymmetric grain boundary.

**Table S3** : Population analysis and magnetism of tungsten atom in Ni GB with comparison with isolated W atom, and for Ni surrounding Ni atoms in GB.

| Species | Ion | s | p | d | f | Total | Charge (e) | Spin (hbar) |
| --- | --- | --- | --- | --- | --- | --- | --- | --- |
| W | 1 | 1.01 | -0.56 | 4.62 | 0.00 | 5.07 | 0.93 | 0.03 |
| W | Iso | 1.00 | 0.00 | 5.00 | 0.00 | 6.00 | 0.00 | 3.00 |
| Ni | 6 | 0.88 | 0.65 | 8.64 | 0.00 | 10.16 | -0.16 | -0.10 |
| Ni | 8 | 0.80 | 0.67 | 8.65 | 0.00 | 10.12 | -0.12 | -0.23 |
| Ni | 17 | 0.82 | 0.65 | 8.65 | 0.00 | 10.12 | -0.12 | 0.20 |
| Ni | 19 | 0.90 | 0.62 | 8.64 | 0.00 | 10.15 | -0.15 | 0.07 |
| Ni | In GB | 0.80 | 0.60 | 8.60 | 0.00 | 10.00 | -0.03 | 0.39 |
| Ni | Iso | 1.25 | 0.00 | 8.75 | 0.00 | 10.00 | 0.00 | 0.60 |

**Table S4** : Population analysis and magnetism of Mn atom in Ni GB with comparison with isolated Mn atom, and for Ni surrounding Ni atoms in GB.

| Species | Ion | s | p | d | f | Total | Charge (e) | Spin (hbar) |
| --- | --- | --- | --- | --- | --- | --- | --- | --- |
| Mn | 1 | 0.62 | 0.86 | 5.24 | 0.00 | 6.71 | 0.29 | 2.51 |
| Mn | 1 | 2.00 | 0.00 | 5.00 | 0.00 | 7.00 | 0.00 | 2.50 |
| Ni | 6 | 0.80 | 0.68 | 8.68 | 0.00 | 10.17 | -0.17 | 0.26 |
| Ni | 8 | 0.76 | 0.62 | 8.66 | 0.00 | 10.04 | -0.04 | 0.29 |
| Ni | 17 | 0.77 | 0.62 | 8.66 | 0.00 | 10.04 | -0.04 | 0.29 |
| Ni | 19 | 0.80 | 0.67 | 8.68 | 0.00 | 10.16 | -0.16 | 0.25 |
| Ni | In GB | 0.8 | 0.6 | 8.6 | 0.00 | 10.00 | -0.03 | 0.39 |
| Ni | Iso | 2.0 | 0.0 | 8.0 | 0.00 | 10.0 | 0.00 | 0.61 |

**References**

[1] Seah M. Grain boundary segregation. Journal of Physics F: Metal Physics. 1980;10:1043.

[2] Všianská M, Šob M. The effect of segregated sp-impurities on grain-boundary and surface structure, magnetism and embrittlement in nickel. Progress in Materials Science. 2011;56:817-40.

[3] Razumovskiy VI, AYL, Razumovskii IM, Ruban AV. Analysis of the alloying system in Ni-base superalloys based on ab initio study of impurity segregation to Ni grainboundary. . AdvMaterRes. 2011;278:192-7.

[4] Yamaguchi M, Shiga M, Kaburaki H. Grain Boundary Decohesion by Impurity Segregation in a Nickel-Sulfur System. Science. 2005;307:393-7.

[5] Clark Stewart J, Segall Matthew D, Pickard Chris J, Hasnip Phil J, Probert Matt IJ, Refson K, et al. First principles methods using CASTEP. Zeitschrift für Kristallographie2005. p. 567.

[6] Goumri-Said S, Ul Haq B, Ahmed R, Abdellatif G, Shaari A, Butt F.K., Kanoun M. B., Dominant ferromagnetic coupling over antiferromagnetic in Ni doped ZnO: First-principles calculations, Front. Phys. (2016) 11: 117101.
